# Touch medicine: bridging the gap between recent insights from touch research and clinical medicine and its special significance for the treatment of affective disorders

**DOI:** 10.3389/fpsyt.2024.1390673

**Published:** 2024-05-22

**Authors:** Francis McGlone, Kerstin Uvnäs Moberg, Henrik Norholt, Michael Eggart, Bruno Müller-Oerlinghausen

**Affiliations:** ^1^ Department of Life Sciences, Manchester Metropolitan University, Manchester, United Kingdom; ^2^ Department of Neuroscience & Biomedical Engineering, School of Science, Aalto University, Helsinki, Finland; ^3^ Department of Animal Environment and Health, Swedish University of Agricultural Sciences, Skara, Sweden; ^4^ SomAffect - The Somatosensory & Affective Neuroscience Group, Liverpool, United Kingdom; ^5^ Faculty of Health Sciences, Brandenburg Medical School Theodor Fontane, Neuruppin, Germany; ^6^ Faculty of Social Work, Health and Nursing, Ravensburg-Weingarten University of Applied Sciences, Weingarten, Germany; ^7^ Faculty of Medicine and Psychology, Brandenburg Medical School Theodor Fontane, Neuruppin, Germany; ^8^ Charité University Medicine Berlin, Berlin, Germany

**Keywords:** touch medicine, affective touch, depression, interoception, oxytocin, CT afferents, stress regulation, massage therapy

## Abstract

Interpersonal touch represents the primal sensory experience between humans, fostering social bonding from the cradle to the death bed. In recent decades “affective touch” has been intensely studied, stimulated by the discovery of a population of mechanosensitive unmyelinated C-tactile afferents in mammalian skin. A lack of touch in childhood is associated with negative consequences for psychosocial and physical health and the benefits of professional touch techniques in the prevention and treatment of various diseases have been shown over and over again in clinical studies. However, its application in mainstream clinical applications remains limited. To bridge the gap between recent discoveries in touch research and clinical medicine, we propose the establishment of a new discipline: ‘Touch Medicine’. Here, we unfold the potential of Touch Medicine by focusing on the treatment of depression, which in our view is primarily a disorder of the lived body. Controlled studies and systematic reviews have demonstrated the antidepressant, anxiolytic and analgesic effects of specific massage techniques. Underlying mechanisms of action are currently under investigation, ranging from interoceptive, endocrinological, to stress-related or psychological underpinnings. Touch Medicine represents a novel interdisciplinary field connected to various medical specialities such as neonatology, pediatrics, pain medicine, neurology, psychiatry, and geriatrics – but also clinical psychology and psychosomatic medicine might benefit from the integration of these findings into their daily practice.

## 1 Introduction

Social touch constitutes a basic human need, conveying empathy, love, care, intimacy, and social belonging (1, 2). The ‘laying on of hands’ as practiced in Judaism was adopted by Christianity with biblical accounts of bringing a formerly sick person back into social life. Without any mechanistic understanding of the observed benefits from such touch practices theories abounded such as their being energy fields (“biofields”) around all living organisms, with disruption in these fields causing disease. Traditional practices such as acupressure and Tui na, as well as practices of more recent origin [such as Therapeutic Touch^®^ (3)], are based in these beliefs. Nonetheless, accounts of miraculous interventions serve as timeless testimonials to the transformative power of compassionate touch. In recent decades, modern scientific approaches have demonstrated unequivocally how prolonged absence of affectionate touch in infancy and childhood causes long-term psychological and physical damage, increasing the risk of disturbed attachment patterns and depression later in life (4–6). Parallel to these findings, experimental and clinical research on the efficacy, as well as the potential mechanisms, of both social and salutary touch has yielded a plethora of clinically relevant insights (e.g., 7–9). Regrettably, these discoveries remain virtually unknown to most physicians. Although modern medicine is becoming increasingly “biological” and body-centered, it lacks an understanding of the distinction between the lived body (“Leib” in German) and the solid objectifiable “corporeal” body (10). This distinction is emphasized in modern embodiment concepts (11), underlying the paradigm of “embodied cognition” in which psychiatry is perceived as relational medicine (12, 13). Hence, in the following we aim to connect findings of modern touch research to clinical medicine. In this context, we will emphasize the potential benefit of salutary touch, based on elements of “affective touch” (14) in the treatment of mental and psychosomatic disorders (15). Exciting potential exists in integrating scientific findings on professional touch techniques including massage therapy in areas like pregnancy (16) and depression into modern medical practice (5, 7, 17). Although our primary focus will be depression, this is only a section of a wide array of potential applications from the beginning of life to its end. In other words, from neonatology all the way through to geriatrics and hospices. Henceforth, we propose the establishment of a new discipline “Touch Medicine”, which makes use of manual touch techniques for the prevention, therapy, rehabilitation, and palliative care of a wide range of mental and physical disorders, but importantly based on recent insights into the neurobiological basis of ‘affective touch’. We are naturally aware that Touch Medicine or “salutary touch” is already practiced in nursing and geriatric care. In the context of depression, we do not consider Touch Medicine to be in a competitive but rather supportive relationship with psychotherapy, targeting directly the somatic dimension of depression. Touch Medicine provides direct access to bodily sensations i.e. to disturbed interoceptive processes or stress-regulating axes via the skin, thus improving mental health (18). A special feature of the practice of Touch Medicine is that the patient can remain in a state of passivity and yet respond to treatment. This may be particularly relevant in the treatment of e.g., chronic fatigue in cancer patients (19).

## 2 Historical outline of touch research

The skin as a mediator of touch is the largest sensory organ in humans, developing from the ectoderm, as does the nervous system, thereby making an ontogenetic link between skin and brain plausible. The sense of touch is the first sense to develop in embryonic development, around the eighth week of pregnancy. Unlike the other senses, a complete loss of the sense of touch in new-born’s has never been reported. A human being can be born deaf or blind, but without the sense of touch it would not be viable. There are rare cases of the loss of the sense of touch in adulthood with neuronopathy patients who, incidentally, have provided essential supporting evidence for the existence of two main touch modalities in humans – a fast myelinated one (selectively lost in neuronopathy) that underlies discriminative touch (i.e., perception of pressure, vibration, slip, and texture), and a recently discovered slow unmyelinated one (preserved in neuronopathy), of prime relevance for this report (14). Important milestones in touch research will be reviewed below (2, 9).

### 2.1 Early observations and experiments

In the mid-twentieth century, the first publications on the significance of touch appeared at an intersection between medicine and developmental psychology. René Spitz (20) recognized the biological necessity of loving care in institutionalized infants and toddlers in nursing homes who, after separation from their mothers, were provided with sufficient food and good hygienic standards, but were rarely touched and held by the staff. As a result, these infants showed high mortality rates and significant behavioral problems such as deprivation syndrome or anaclitic depression. Similar consequences of touch deprivation were later also described in Romanian orphans (1). Important insights into the significance of touch in early life were gained through the experiments of Harlow, who used artificial dummies to show in newborn rhesus monkeys isolated from their mothers that satisfying the need for closeness and physical contact is as important as satisfying the feeling of hunger. As a result of these experiments, the importance of physical contact was recognized by Bowlby and his collaborator Ainsworth as an essential basis for attachment behavior in humans (5, 21).

Numerous mammalian and human studies bear witness to the negative consequences of deficient caregiver early touch provision (22–26). Following Hofer’s groundbreaking research (22), several experimental studies in rodents investigated the effects of the quantitatively different ways in which mothers touch and lick their offspring- a behavior similar to “affective touch” in humans (see below).

Prematurely born human infants provided with maternal skin-to-skin contact (“Kangaroo Mother Care”) demonstrate improved cardiovascular and respiratory stability (27), improved autonomic regulation, immune system function (28), and enhanced cognitive development compared to standard incubator care (29–31). Here again we see a putative link between the absence of tactile stimulation the developing fetus would have been exposed to *in utero* when placed in an incubator, and the reported ~30% increased risk of autism (32) in preterm babies. The positive developmental sequalae of enhanced early touch experiences have been borne out in controlled longitudinal studies demonstrating at age 10 years and in early adulthood, improved executive function, more organized sleep patterns, attenuated stress response, and improved mother-child reciprocity (33, 34).

Full-term neonates also benefit from increased maternal skin-to-skin contact, supporting the seminal research findings of Ainsworth et al. (35) and Main (36, 37) showing, for example, less angriness/aggression and fostering a secure attachment style. Bigelow and Power (38) showed advanced socioemotional development in 3-month-old infants of mothers engaging in daily maternal-infant skin-to-skin contact during the first postpartum month. A follow-up study at age 9 years provides further evidence for the robust positive long-term developmental effects of early enhanced touch provision on social behavior (39).

### 2.2 Discovery of C-tactile afferents

In the context of this article we will place special emphasis on studies that focused not only on the association between the type and/or intensity/frequency of early physical contact, attachment, and the risk of later mental disorders such as depression (33, 40–45), but, importantly, recent research into the neurobiological mechanisms hypothesized to contribute to these associations. Sufficient evidence has been accumulated to support the hypothesis that ‘early physical contact represents a primary preventive intervention with positive effects on physical and psychosocial health’. For a long time the idea had prevailed that touch stimuli were only transmitted to the CNS via myelinated, fast-conducting, low-threshold mechanoreceptors (LTM) - Aβ afferents and the dominant focus of this research were LTMs populating the glabrous skin of the hand with the exquisite sensitivity of the digits seen as the ‘fovea’ of the somatosensory system. However, a major turning point in touch research over the past 30 years has been the discovery of a system of mechanosensitive unmyelinated nerves innervating the skin of the whole body, called C-low threshold mechanoreceptors (C-LTMRs) (14, 46) or C-tactile afferents (CT). CTs are a structurally and functionally independent group of low-threshold mechanoreceptors afferents, first discovered in the cat saphenous nerve by a Swedish neurophysiologist, Yngve Zotterman, in 1939 (47) and subsequently in humans by other Swedish researchers in the late-1980s (48). CTs respond to touch qualities corresponding to tender touch (e.g. stroking, caressing, gentle touch). Under experimental conditions using the single-unit nerve recording technique of microneurography (46) it was found that CTs show the strongest firing rate at stroking speeds between 1–10 cm/s with the application of gentle pressure and a temperature optimum corresponding to the skin temperature (49). This electrophysiological velocity tuning of CTs was mirrored in psychophysical studies where participants reported brush stroking velocities of 1–10 cm/sec as being more pleasant than slower or faster velocities (50). Psychophysical studies have also provided insight into the inferred topographical innervation density of CTs across the body with pleasantness ratings reported as higher from proximal sites, and in particular body sites that are inaccessible to ego-grooming such as the shoulder and back (51) – touch to these body sites will be perceived as more rewarding. This raises an interesting evolutionary explanation when we look at primate grooming, as an inability to ego- groom the back requires soliciting an allo-groomer to carry out this function – the beginning of social grooming? On the psychological level, the activation of CTs is accompanied by a subtle feeling of well-being which, from a perceptual-psychological point of view, cannot be attributed to an exteroceptive but to an interoceptive source i.e. an inner, pleasant body feeling; see below (52). These touch qualities are often referred to as *affective touch* - interestingly, this term was first used by nursing scientists to distinguish them from non-task-specific touch (53), and has recently been reviewed by Schirmer et al. (54). fMRI findings showed that optimal stimulation of CT afferents activates cortical limbic regions in particular, including dorsal posterior insula, posterior sulcus temporalis superior, medial prefrontal cortex, dorsal anterior cingulate cortex (55). Processing of CT-associated touch shows overlap with centers also involved in processing emotions and social cognitions (14, 56). This could have clinical consequences, particularly with regard to psychiatric and psychosomatic disciplines (57). Apart from the neuronal “bottom-up” processes, top-down mechanisms are also involved in the processing and perception of touch stimuli. In particular, contextual “top-down” factors determine the quality of the touch experience; its meaning for the receiver depends not only on the person touching, but also on the underlying intention (“why is touching taking place?”) (58, 59). In other words: a physically defined stimulus, e.g. the gentle touching of the back of the hand, has a completely different meaning when occurring in the context of a neurological examination or a nascent erotic contact.

## 3 Professional touch in the treatment of depression

### 3.1 Conceptual approach

Although psychotherapy proves to be effective in many cases, two meta-analyses suggest a rather restricted overall effectiveness of the psychotherapeutic approach in depression (60, 61). Unfortunately, also the average effectiveness of antidepressive treatments by medication following international guideline recommendations is unsatisfactory (62–64). This limitation also applies to the currently strongly marketed mindfulness techniques (65). About one third of therapy responders still complain about residual symptoms such as anxious states, sleep disorders etc. (66). These findings underline the need for further complementary treatment of depression. A recent study also showed that physical activation through emotional stimuli is reduced under the impact of antidepressant medication (67). This is consistent with findings from a pharmacological fMRI study that suggests dampening effects of the antidepressant escitalopram on insula activity during the processing of stimuli with positive or negative connotation (68). The search for further complementary or integrative therapeutic options thus seems justified.

Depression is primarily characterized by anhedonia, i.e. the inability to perceive positive stimuli as such; and this refers not only to the cognitive level, but to the entire sensorium. In modern, highly formalized psychiatric diagnostics, this fundamental condition is almost lost in an internationally consented list of symptoms. It is also not yet understood in which way pharmacological treatment can positively influence the primary anhedonic condition. Depression is a disorder affecting the whole person, affecting the lived body as well as the affective and cognitive levels. The rigidity and constriction that overwhelmingly and threateningly surround the depressed person affects their physical as well as mental functions, which is also expressed, for example, in the altered experience of time implying the strong dominance of the past (69). The depressive state is not “accompanied” by various “somatic symptoms” as it is often said (70), but the totally changed somatic experience represents a core symptom of depression, so that one could also speak of somatopsychic (instead of psychosomatic) syndromes (71). The lived body of the depressive patient is sick. In this context, Thomas Fuchs refers to the “corporealization” of the body, i.e. depressed patients are no longer at home in their body, which is experienced as a resistant body (13) [for a respectful critique of this position, ref (72)].

The subjective body experience in depression has been studied systematically much less often than in schizophrenia. Yet a small number of studies illustrate that depressive individuals clearly feel their altered corporeality and evaluate it negatively (73, 74). A new phenomenological-qualitative study also indicates that those affected describe their bodily sensations as blockage, heaviness, emptiness, alienation, or paralysis in the head or in the whole body. Constricting, pressing feelings in the chest and the abdominal area are associated with specified emotions by some study participants (67).

Many forms of physical pain, chest tightness, dizziness, palpitations, loss of appetite and libido, shivers, hot flushes, stomach pressure, hypothermia and cramping of the limbs etc. can be observed in depressed patients. As early as 1966, muscular contractions in the neck and back of depressed patients were reported by Paul Kielholz, a Swiss psychiatrist (75). We are aware of the comment of a female patient who described her experience as follows: “I contracted more and more inside, even got physical pain from the deformation and clenching “. Another commented: “The disharmony between head and body - as if the body was getting lost because the head was so stressed…”.

Considering the anhedonic state affecting the entire sensorium a lack of continuous, structured “mini-arousal” could be hypothesized. In fact, a cortical hypofunction during emotional arousal has been found in depressed patients (76). Thus it might be speculated that a specific “psychoactive” massage therapy in an atmosphere of attentiveness and empathy could cause a subtle arousal. In fact Ackerley et al. (77) observed the triggering of an ultra-late EEG potential in probands having received affective touch. Under the condition of repeated soft touch we may then expect also increased synaptic interconnectivity (78, 79).

### 3.2 Findings from clinical studies

In the former psychiatric hospital of the university of Basel (Switzerland) all depressed patients received neck massage (75) based on the doctoral thesis and experimental study by Maurer-Groeli (80). In addition, the rewarming time of the hands after exposure to cold was routinely measured as an indicator of therapeutic success.

In 2004, the clinical psychologist Moyer and colleagues presented the first meta-analysis of the publications available at that time on the antidepressant and anxiolytic efficacy of massage therapy, concluding that its effect size was equivalent to that of psychotherapy for the indications under study (81). Baumgart et al. (82) surveyed 22 RCTs on patients with depression or anxiety disorders, selected according to strict criteria, and published up to 2009. They found that in the majority of cases there was a clear superiority of massage therapy over the various control conditions, although the authors felt that the heterogeneity of the latter stood in the way of conducting a meta-analysis. In some studies long-term effects were also reported and overall, in agreement with Moyer et al. (81), more significant effects were found in studies on depressive patients than in those with an anxiety disorder. A more recent randomized-controlled study, however, suggests that the therapeutic effects of massage therapy in generalized anxiety disorder have so far been underestimated (83). The meta-analysis by Hou, which however is nearly exclusively based on studies by Tiffany Field and colleagues also reported a statistically significant antidepressive effect of massage therapy (84). However, the reviewers suggest standardized protocols, various depression rating scales and target populations in further studies [ref. also (57)]. An earlier systematic review concluded that the evidence for efficacy of massage therapy was not sufficient at the time of the review (85). In the following, we shall refer to three randomized-controlled studies which have been selected because all of them applied individual modifications of the psychoactive massage technique (see below).

The results of one of the first RCTs on hospitalized depressive patients as well as healthy volunteers proved a clear superiority of Slow Stroke^®^ Massage (full body massage, mostly effleurage techniques with low pressure, 60 minutes) compared to a control condition without touch (86). More recently, Baumgart et al. (87) demonstrated superior analgesic and antidepressant efficacy of the “psychoregulative massage” which they developed for patients with psychosomatic back pain. The sustainability of the effects over 3 months was particularly remarkable (87). Tiffany Field, director of the Touch Research Institute in Miami (USA), was also able to prove the antidepressant and analgesic effect of somewhat different modes of massage in several earlier studies (7). Another controlled study from the University of Wuerzburg (Germany) in depressed outpatients also showed a clear superiority of “Affect-Regulating Massage” compared to a standardized relaxation method (88). The “psychoactive” massage techniques used in some of the cited studies (87–89) are mostly based on “effleurage” i.e. gentle stroking or in other words by using essential elements of affective touch; their target organ thus is predominantly the skin and the fascia superficialis. Some elements of “petrissage” might be added particularly by slightly increased pressure. The duration of the massage sessions was mostly 50–60 minutes applied over a frequency of 4–8 sessions with 1–2 sessions weekly. The therapeutic aim is not just relaxation or sedation but counteracting the disturbed interoceptive processes (see below) and amelioration of the total psychophysical burden found in depressed patients.

The specific methodological requirements to be observed in future studies were addressed in a recent editorial stressing particularly a) the need for accurate description of the massage technique used, b) a suitable control condition in randomized trials, c) the use of standardized observer and self-rating scales, and d) a therapist consistency in experimental and control groups (57). Even if for methodological reasons some questions remain currently unanswered, compelling evidence in empirical touch medicine research has nevertheless been reached which, in our opinion and strongly supported by Tiffany Field a quarter of a century ago (7) would justify the introduction of this treatment option in psychiatric or psychosomatic clinics.

### 3.3 Potential mechanisms of action

Certainly, there is no “one” mechanism of action for the described antidepressive and analgesic effects of massage, which, by the way, have also been repeatedly demonstrated in patients with cancer complaining of e.g. chronic pain or fatigue after chemotherapy or surgical intervention (90, 91). They have to be discussed on different explanatory levels such as on the background of neurophysiological or psychological constructs, but also importantly on the immunological level. Some of these have been alluded to in the foregoing sections. Certainly, the activation of the CT afferents and their connection to the interoceptive system plays an important role in this context (92). However, there exist also important hormonal factors, of which the oxytocinergic effects are of particular importance. Various effects of psychoactive massage therapy mostly based on “affective touch” (14, 93) are very probably mediated via the oxytocinergic system; this is especially true for their proven antidepressant and analgesic effects (see above) (94, 95). Therefore, the oxytocin system will be discussed in more detail below.

#### 3.3.1 Psychological and neurophysiological mechanisms

From a psychological standpoint interpersonal (social) touch can be regarded as a non-verbal exchange of affection supporting the development of affiliative bonds, enhancing social cognition, and facilitating emotion regulation (58, 96, 97). Additionally, touch serves as a conduit for conveying various emotions even in the absence of vocal expression within social interactions (98). Research has consistently demonstrated that interpersonal exchanges of affection is associated with physical, psychological, and social health benefits (99, 100).

In this context, the psychosocial effects of affective touch must be emphasized. For instance, social touch has been found to mitigate the adverse effects of social exclusion (101) – so often burdening depressed patients who lack meaningful connections with their social environment and suffer from loneliness, which impedes recovery from their disease (102). Besides, these findings demonstrate that affective touch not only alleviates physical pain, but also attenuates social pain, possibly due to shared neural representations underlying these systems (103). Preliminary findings also underscore the crucial role of interpersonal touch in mitigating feelings of loneliness (104).

Furthermore, extensive literature documents the stress-buffering effects of social support paralleling the reduced stress-reactivity of the hypothalamic-pituitary-adrenal (HPA) axis and the autonomic nervous system (105). Affective touch may facilitate an adaptive response to acute stressors by both regulating the physiological condition of the body and promoting stress resilience (106–108). Therefore, considering the close link between positive body contact, the development of secure attachment (109) and adaptive stress regulation from infancy through adulthood, the clinical application of affective touch in order to promote the body’s regenerative mechanisms against stress (110) may offer promising avenues for the treatment of affective or other stress-related disorders.

#### 3.3.2 Oxytocinergic system

The cerebral oxytocin neurons originate in the hypothalamic supraoptic and paraventricular nerve (PVN). They project to the posterior pituitary gland, from where oxytocin is released into the blood circulation. Upon strong stimulation, dendrites and nuclei of these neurons also release oxytocin intracerebrally (111). Furthermore, special oxytocinergic neurons project from the PVN to various regulatory brain areas, e.g. locus coeruleus, periaqueductal cavernous grey, raphe nuclei, etc., which have important autonomic-regulatory functions (112). Axon collaterals of the neurons that draw to the pituitary gland also reach the amygdala, insula, and cortex (113, 114).

This complex system leads to a variety of physiological reactions through the release of oxytocin both into the bloodstream and through the influence of central transmitters: in the foreground are the stimulation of prosocial behavior, the reduction of anxiety and stress levels, the promotion of calm and well-being, analgesic, and anti-inflammatory effects, but also the triggering of regenerative processes (95).

The oxytocin system is activated in part or in whole in different situations, e.g. triggered by different types of social interaction (94, 95, 115). This can also occur under somatosensory stimulation such as the birth process or breastfeeding (116, 117). But also the activation of cutaneous afferents, especially through gentle stroking, stimulates the release of oxytocin (118, 119). It is not known exactly which cutaneous afferents activate the oxytocin-producing neurons. Most likely, however, the C-tactile afferents described in more detail above also play an important role (120). The close skin-to-skin contact between mother and newborn infant that is often practiced nowadays implying also static pressure together with warmth results in a remarkably strong release of oxytocin. This observation suggests an important role of additional cutaneous receptors for the hormonal response (121).

Since gentle skin touch causes an oxytocin release in humans, with subsequent activation of serotonergic raphe nuclei as well as dopaminergic neurons in the striatum and nucleus accumbens, the neuroscientific level of explanation also suggests that e.g. gentle massages have a positive influence on the mood (122, 123). The activation of the oxytocinergic system is likely to play a significant role in this context (94, 95, 124, 125).

Incidentally, attempts have also been made, albeit largely unsuccessfully, to produce antidepressant effects through intranasally applied oxytocin. This negative result was used as evidence for the non-existence of an antidepressant effect of oxytocin (94, 114). However, this conclusion is not compelling because exogenously administered oxytocin does not have the same effect as when it is released due to the activation of certain cutaneous afferents. A gentle touch on the skin, as in a psychoactive massage, activates those components of the intracerebral oxytocinergic system that are responsible for the anxiolytic, analgesic, relaxing and antidepressant effects mentioned above (95, 125). A physiological activation of the oxytocin system leads to high concentrations of the hormone at specific nerve endings and receptors and not, as in the case of intranasal oxytocin application, in unspecified, ubiquitous brain regions. Against the background of such diverse oxytocin effects, the end effect of intranasal delivery is likely to be the sum of partly contradictory individual effects of the hormone. In contrast, the targeted activation of certain skin receptors and the resulting release of oxytocin through e.g. an affective-touch-based massage will trigger a very specific pattern of effects, e.g. in the form of an antidepressant effect.

#### 3.3.3 Interoceptive system

Another level of explanation is based on the sensory modality of interoception including all bodily sensations that relate to the physiological state of the entire body - i.e. visceral sensations and stimuli from thermo-, chemo-, nociceptors as well as low-threshold mechanoreceptors of the touch system i.e. the CTs (126). The insula plays a central role in the integration of all individual sensations into a bodily self (“material me”) (127). The term interoception was introduced by Sherrington to distinguish it from exteroception and proprioception (128). It is related to the coenaesthesia (“Gemeingefühl”) described by German physiologists less than a century ago (129). Beyond homeostatic regulatory mechanisms, more recent findings suggest an influence of interoception on affect, cognition, and behavior (18).

Interoception research thus contributes significantly to the pathogenesis of affective disorders, for example by confirming central assumptions of ancient theories of emotion. Thus, William James had already postulated towards the end of the 19th century that specific visceral changes occur in response to a stimulus, which in turn are perceived as an emotion (130). Accordingly, in a systematic analysis evidence was found that patients with moderate depression are poor cardiac perceivers - i.e. they significantly underestimate the number of their heartbeats within defined time intervals compared to healthy controls, indicating a dampened cardiac interoception. This ability is related in a complex manner to affective and cognitive symptoms of depression, e.g. affective flattening or decision-making difficulties (131). Maladaptive attentional styles towards interoceptive stimuli, such as worry about unpleasant bodily sensations, reduced body confidence and disturbed interoceptive self-regulatory mechanisms, are characteristic symptoms of depression and predict response to treatment in hospitalized patients suffering from depression (132, 133). Interoceptive impairments have also been found in other mental health conditions including eating, anxiety, addictive and somatic symptom disorders (18). In a review of randomized controlled trials, changes in body sensations were consistently associated with clinical improvements suggesting an interoceptive mechanism of action (134).

Thus, if massage therapy can counteract depressive anhedonia (see above), it can be surmised that its clinical effects are mediated through the interoceptive system (92, 135). Preliminary results suggest that interoceptive states can be influenced via the skin, e.g. via a gentle touch-mediated improved accuracy of heartbeat perception (136, 137). In the foregoing chapters we emphasized that the preferred stimulus for CTs is a low force and velocity touch delivered at skin temperature. For the sake of completeness, however, it should be mentioned that under certain conditions, which still must be defined more precisely, stronger pressure can also achieve positive effects (138) which may be traced back to the manipulation of myofascial tissue (139). In this context, Field et al. (140) underline the importance of vagal activation which indeed plays a significant role in some interoceptive mechanisms (18). A recent study also suggests that the stress-reducing and relaxing effect of a standardized massage intervention is accompanied by an increased activity of the parasympathetic nervous system, which was shown by a significant increase in heart rate variability (110). The latter observations are in fact consistent with activation of the central oxytocinergic system, which exerts prominent effects on vagal nerve function via projections to the dorsal vagal motor nucleus (DMX) and the nucleus tractus solitarius (NTS) (119). These findings are important for the treatment of affective disorders, as depression is associated with reduced heart rate variability, which is predictive of cardiovascular events (141).

## 4 Conclusions and future directions

The effectiveness and safety of touch therapy can be regarded as sufficiently proven within a broad spectrum of medical indications from neonatology to geriatrics and palliative medicine, especially also in the field of psychiatry/psychosomatics and clinical psychology. The studies performed in patients with mental disorders uniformly show a decrease of depressive mood and anhedonia as well as unrest and bodily complaints including pain without the risk of adverse effects. Nevertheless we are still in need of further clinical studies particularly focusing also on long-term effects and respecting modern methodological requirements (57). Widely discussed mechanisms of action refer to the hormonal, particularly oxytocinergic effects, the positive influence on the HPA-axis and on interoceptive processes. Particularly the soft stroking technique of “psychoactive” massage, i.e. affective touch, activates limbic brain areas via C-tactile afferents. Research over the past ~30 years thus provides evidence-based support for a paradigm shift in the non-pharmacological and psychotherapeutic treatment of affective disorders. The effectiveness and practicability of Touch Medicine treatments need to be tested now on a larger scale and hopefully with the support of the health insurance companies. In the area of nursing care, the already existing positive experiences on the effectiveness of salutary touch, e.g. in dementia (142) and neonatology (124) should be expanded and confirmed by appropriate studies. Furthermore, it seems urgent that the existing empirical findings of Touch Medicine in various medical disciplines should be integrated into national and international guidelines.

## Author contributions

FM: Writing – review & editing, Writing – original draft, Conceptualization. KM: Writing – review & editing, Writing – original draft, Conceptualization. HN: Writing – review & editing, Writing – original draft, Conceptualization. ME: Writing – review & editing, Writing – original draft, Conceptualization. BM: Writing – review & editing, Writing – original draft, Project administration, Conceptualization.
